# Correction to: Exosome-transmitted miR-128-3p increase chemosensitivity of oxaliplatin-resistant colorectal cancer

**DOI:** 10.1186/s12943-020-01211-8

**Published:** 2020-05-12

**Authors:** Tong Liu, Xin Zhang, Lutao Du, Yunshan Wang, Xiaoming Liu, Hui Tian, Lili Wang, Peilong Li, Yinghui Zhao, Weili Duan, Yujiao Xie, Zhaowei Sun, Chuanxin Wang

**Affiliations:** 1 Department of Clinical Laboratory, The Second Hospital of Shandong University, No. 247 Beiyuan Street, Jinan, 250033 China; 2 Department of Clinical Laboratory, Qilu Hospital, Shandong University, Jinan, 250012 Shandong Province China; 3 Department of Preventive Medicine, Shandong Provincial Traditional Chinese Medical Hospital, Jinan, 250012 People’s Republic of China; 4 Cancer Center, Qilu Hospital, Shandong University, Jinan, 250012 Shandong Province China; 5 Department of Surgery, The Affiliated Hospital of Medical College Qingdao University, Qingdao, 266071 Shandong Province China

**Correction to: Mol Cancer (2019) 18:43**

**Fig. 1:**
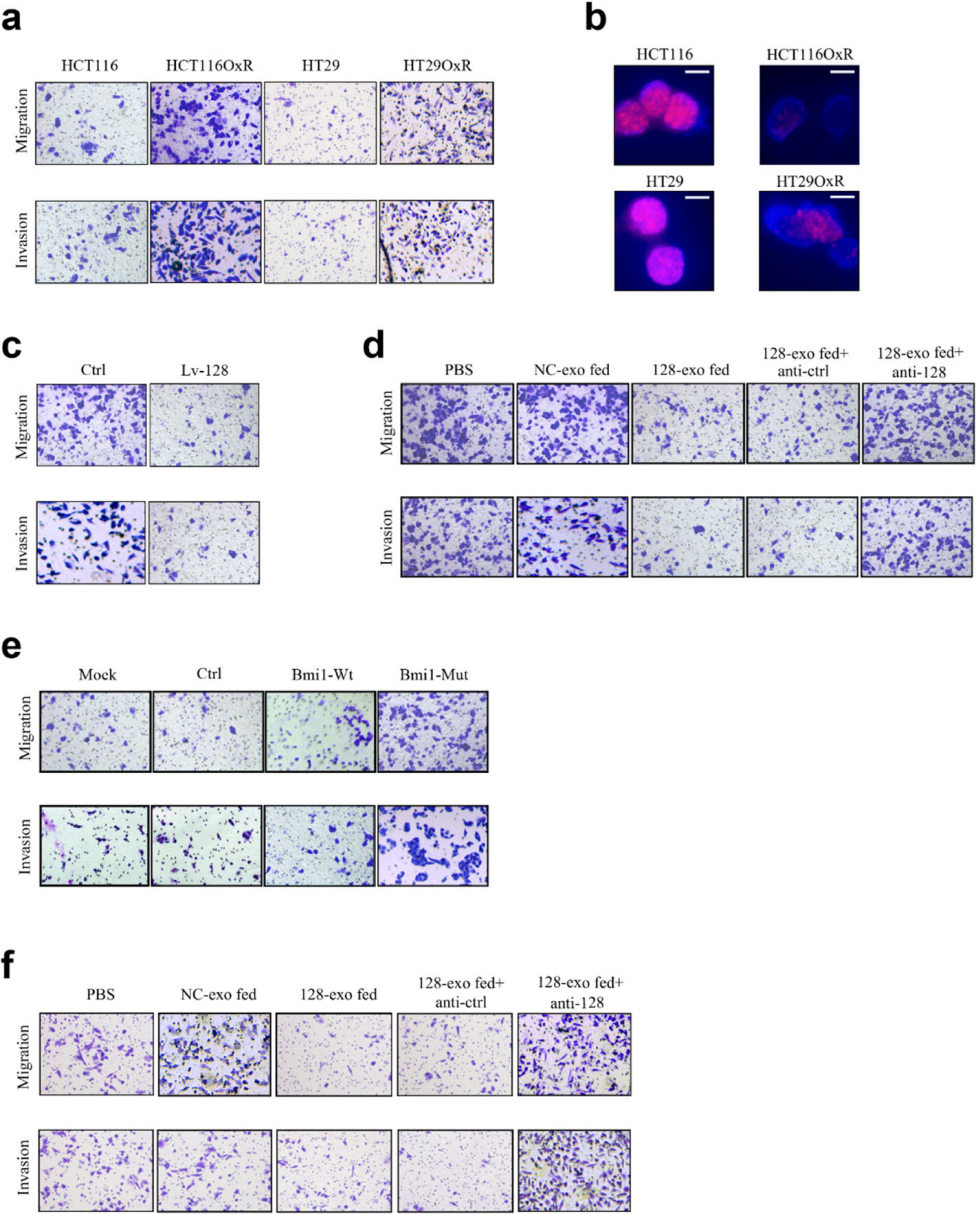
**a.** Migration and invasion ability of parental and resistant CRC cells were assessed by Transwell assay (replace Fig. 1e). **b.** The immunofluorescence analysis of nuclear foci for γ-H_2_AX expression induced by oxaliplatin in parental and resistant cells after 24 h oxaliplatin exposure. Scale bars, 10 μm (replace Fig. 1j). **c.** Migration and invasion ability of HCT116OxR cells transfected with Lv-128 and Ctrl were assessed by Transwell assay (replace Fig. 2f). **d.** Migration and invasion ability of HCT116OxR cells after incubated with indicated factors for 48 h were assessed by Transwell assays (replace Fig. 5d). **e.** Migration and invasion ability of Lv-128 transfected HCT116OxR cells in different conditions were assessed by Transwell assays (replace Fig. 6g). **f.** Migration and invasion ability of HT29OxR cells after incubated with indicated factors for 48 h were assessed by Transwell assays (replace Fig. S4D)

**https://doi.org/10.1186/s12943-019-0981-7**

Following the publication of the original article [1], the authors noticed some incorrect information are shown. Inverted-microscope pictures of transwell assay were placed in the mistaken figure inadvertently and the fluorescence-microscope picture was mistakenly switched. The correct version of figure is given in Fig. 1. The authors apologize for any inconvenience caused, and these corrections do not affect the findings or conclusions of this research.
